# Breakthrough in Silicon Photonics Technology in Telecommunications, Biosensing, and Gas Sensing

**DOI:** 10.3390/mi14081637

**Published:** 2023-08-19

**Authors:** Muhammad Shahbaz, Muhammad A. Butt, Ryszard Piramidowicz

**Affiliations:** Institute of Microelectronics and Optoelectronics, Warsaw University of Technology, Koszykowa 75, 00-662 Warszawa, Poland

**Keywords:** silicon, integrated optics, waveguide, telecommunication, biosensing, gas sensing

## Abstract

Silicon photonics has been an area of active research and development. Researchers have been working on enhancing the integration density and intricacy of silicon photonic circuits. This involves the development of advanced fabrication techniques and novel designs to enable more functionalities on a single chip, leading to higher performance and more efficient systems. In this review, we aim to provide a brief overview of the recent advancements in silicon photonic devices employed for telecommunication and sensing (biosensing and gas sensing) applications.

## 1. Introduction

Silicon (Si) photonics is a groundbreaking technology that merges the fields of Si microelectronics and photonics to enable the manipulation and transmission of light on a Si chip. It leverages the exceptional properties of Si, such as its high refractive index and compatibility with existing electronic manufacturing processes, to create compact and highly efficient optical devices. Si photonics has the potential to revolutionize various domains, including telecommunications, data centers, sensing, and biomedical applications [1,2,3,4,5], by offering high-speed data transmission, low power use, and integration with electronic systems. Its ability to seamlessly integrate photonics with Si electronics opens new avenues for the development of advanced, scalable, and cost-effective solutions for a wide range of applications.

Si, the second most abundant element on Earth after oxygen, possesses exceptional qualities that make it highly suitable for various applications. Its simple cubic crystal structure allows production of defect-free wafers with remarkable purity. Additionally, Si’s thermal conductivity, hardness, and low density are advantageous in semiconductor devices. At a specific wavelength, Si demonstrates a high refractive index and remains transparent to infrared light. The high refractive index of Si allows the miniaturization of devices to incredibly small scales. Moreover, the well-established techniques used in semiconductor processing can be easily applied to Si photonics, enabling cost-effective mass production. Another advantage of Si is its high-quality native oxide, which offers superior characteristics compared with other semiconductors. The oxide serves as an excellent material for Si waveguide (WG) cladding and can host rare-earth dopants for integrated circuits. By controlling the oxide cladding layer, it becomes possible to manipulate light propagation within Si WGs. These properties collectively position Si photonics as a promising solution for integrating photonic circuits.

Si photonics integrated circuits rely on key components to manipulate and control light signals. WGs guide light through total internal reflection, whereas modulators alter light intensity or phase. Photodetectors convert optical signals to electrical ones, and filters selectively transmit specific wavelengths [6,7]. Splitters and couplers distribute and combine signals, and optical amplifiers enhance weak signals. These building blocks enable applications in optical communication, data centers, sensing, and biomedical imaging, propelling advancements in photonics technology [8].

Researchers have made substantial progress in increasing the sensitivity and limit of detection (LoD) of Si photonic sensors. By enhancing the design of WGs and resonators and incorporating advanced materials, these sensors can now detect even smaller quantities of substances, making them suitable for a wider range of applications, including environmental monitoring, healthcare, and security. The integration of sensors on a single Si chip offers numerous benefits, including reduced size, increased portability, and cost-effectiveness. The ability to combine multiple sensors on a single chip allows for compact and robust sensing platforms that can be easily mass-produced. Moreover, advancements in fabrication techniques, such as nanofabrication and complementary metal oxide semiconductor (CMOS)-compatible processes, have enabled the cost-effective mass production of Si photonic sensors [9]. This scalability has opened new opportunities for commercial applications and broader adoption of the technology.

Although Si photonics offers numerous advantages, it is worth noting that other optical platforms, such as indium phosphide (InP) [10], gallium arsenide (GaAs) [11], lithium niobate (LiNbO_3_) [12,13], and rubidium titanyl phosphate (RTP) [14,15], also have their own unique strengths and are preferred for certain specialized applications. The choice of platform depends on factors such as performance requirements, cost considerations, and the specific needs of the application at hand. For more detailed information on optical platforms and fabrication methods, we recommend readers to see [16].

**Figure 1:**
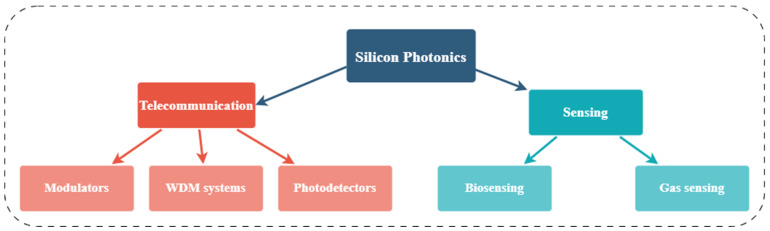
Different applications of Si photonics discussed in this review.

Si photonics remains an evolving field, continuously pushing the boundaries of optical technology. Several review papers have delved into the diverse facets of Si photonics, shedding light on its current state and prospects [17,18,19]. In this review, we delve into the early research efforts and provide an overview of the significant advancements in the field of Si photonics. We emphasize the critical breakthroughs that have shaped the development of this technology. Furthermore, we explore the diverse range of applications (as shown in Figure 1) that leverage Si photonics, showcasing its versatility and potential impact in various fields.

## 2. History

Since the 1970s, researchers have envisioned an optical super chip with integrated optical components [20]. Early studies focused on ferroelectric materials such as lithium niobate (LiNbO_3_) and III-V semiconductors such as gallium arsenide (GaAs) and indium phosphide (InP). LiNbO_3_ stood out for its significant electrooptic coefficient, enabling optical modulation through the Pockels effect. Meanwhile, the III-V compounds offered advantages in terms of laser fabrication, optical amplification, and electronic integration. Si’s widespread use as the preferred semiconductor in electronics prompted researchers to explore the possibilities of Si photonic circuits. This was mainly driven by the potential benefits of integrating photonics and electronics cost effectively. Si has a high refractive index compared with other common materials used in photonics. This property allows for strong light confinement and efficient light guiding within Si WGs and resonators. Consequently, Si photonic components can be designed on a small scale due to the strong confinement of light within Si WGs. This compactness is especially advantageous for on-chip integration and the creation of complex optical circuits. The investigation of Si photonic circuits started in the mid-1980s and has been ongoing since then. A significant volume of research has been conducted on various materials such as SiON [21,22,23,24,25,26,27], Si_3_N_4_ [28,29,30], SiGe [31,32,33,34], SiO_2_ [35,36,37], and SiC [38,39,40]. These materials have been explored concerning their compatibility with standard CMOS technology. Si dominates as the primary semiconductor material for electronics due to its affordability, well-understood properties, and optical confinement capabilities. However, its indirect bandgap limits its effectiveness as a light-emitting material. Researchers have thus sought ways to modify Si’s structure for light emission [41].

**Figure 2:**
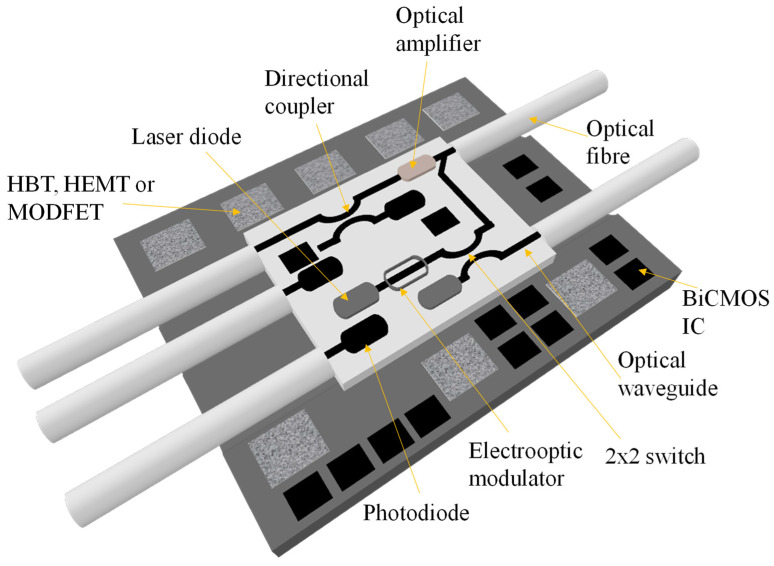
Si-based super chip. Inspired by [42].

Additional efforts were undertaken to define a completely integrated monolithic optoelectronic super chip for Si hybrid integration. This idea was later modified to introduce a hybrid device that relied on a Si platform, incorporating Si optical WGs [42] as depicted in Figure 2.

## 3. CMOS Fabrication

CMOS is a semiconductor technology commonly used in electronic devices and integrated circuits and is crucial for Si photonics, enabling the integration of photonic and electronic elements on a single Si chip. CMOS technology is widely adopted for its energy efficiency, scalability, and versatility, making it a fundamental component in the design and manufacture of modern electronics [16]. Photolithography, using optical or extreme ultraviolet (EUV) light, transfers patterns onto a Si wafer coated with photosensitive material. Ultraviolet lithography (UVL) represents a distinct form of photolithography wherein UV light serves as the exposure source [43]. This technique finds extensive application in semiconductor manufacturing and various industries to produce patterns on a substrate. The utilization of UV light with its shorter wavelength enhances resolution and facilitates the creation of smaller, complex devices [44]. Nonetheless, UVL demands meticulous attention and specialized equipment due to its distinctive properties. Electron beam lithography (EBL) is a potent nanofabrication technique that achieves extremely high-resolution patterns in nanometers, enabling precise nanostructures and devices [45]. Though versatile, it is relatively slow and expensive compared with other lithography methods due to the time-consuming electron beam scanning process [46]. Nanoimprint lithography (NIL) offers a cost-effective alternative [47] to high-resolution lithography techniques such as EBL. It enables high-throughput production by processing large substrate areas simultaneously [48]. Additionally, unlike EBL, NIL does not necessitate complex and expensive equipment.

Reactive ion etching (RIE) [49,50] and chemical wet etching [51,52] are common techniques in microfabrication and semiconductor manufacturing. They offer selective material removal from a substrate for pattern creation. Chemical wet etching suits simple and large-area patterning, whereas RIE is better for high-resolution and anisotropic etching [53].

The combination of CMOS technology with various lithography and etching techniques enables the creation of complex and high-performance photonics devices that drive technological advancements in a wide range of industries. As technology continues to evolve, further innovations in CMOS and lithography techniques will likely contribute to even more exciting developments in the field of photonics and beyond.

## 4. Recent Advances in Si Photonics for Telecommunication

Recent progresses in Si photonics have greatly impacted the field of telecommunications. It combines the advantages of Si-based electronic circuits with the speed and bandwidth of optical communications, enabling the development of highly efficient and scalable optical devices [54,55]. By integrating optical components such as modulators, detectors, and WGs directly onto a Si chip, Si photonics allows for the seamless integration of optical and electronic functionalities. This technology enables high-speed data transmission over long distances, facilitates the growth of data-intensive applications, and provides a pathway for the development of compact, low-cost, and energy-efficient telecommunication systems. Its compatibility with existing Si manufacturing processes makes it an attractive solution for driving the next generation of communication networks, data centers, and high-performance computing infrastructures [56]. In the following sections, we discuss various technologies and applications associated with Si photonics.

### 4.1. Si-Based Modulators

In recent times, there has been a remarkable advancement in the efficiency of Si optical modulators, which corresponds to substantial research endeavors conducted by academic institutions and businesses on a global scale [57]. The objective behind enhancing the performance of optical modulators in Si is evident. These modulators play a pivotal role in most photonics systems used in data communication applications. By utilizing Si photonics as a cost-effective foundation for constructing these systems, optical-based communication becomes a viable option for numerous short-distance connections. Over the years, numerous methods have been explored to achieve modulation in Si by integrating it with emerging optical materials such as graphene [58,59,60,61]. Chip-scale modulation is an area of great interest, particularly concerning silicon-on-insulator (SOI) modulators [19].

Si modulators employ the phenomenon known as free-carrier plasma dispersion. In the context of Si photonics devices, implanted dopants are used to achieve optical modulation in terms of phase and amplitude. This modulation is accomplished by inducing changes in the complex refractive index of a material containing an excess of either electrons or holes, which is wavelength dependent. Consequently, by manipulating the concentration of free carriers within a WG [62], it becomes possible to modulate the phase and amplitude of the light propagating through it.

Si modulators can be integrated with CMOS technology, which is widely used in the semiconductor industry. This integration enables the creation of photonic integrated circuits that combine both electronic and photonic components on the same chip. This compatibility allows for cost-effective mass production and easy integration with existing electronic systems. These modulators can be fabricated on a small scale, enabling the creation of compact devices and circuits. This compactness is especially valuable for applications where space is limited, such as in data centers, optical interconnects, and portable devices. Moreover, Si modulators are capable of achieving high modulation speeds, making them suitable for high-speed optical communication systems. They can operate at data rates exceeding tens of gigabits per second and are continuously being optimized for even higher speeds [63].

However, Si modulators can exhibit nonlinear behavior, which can lead to signal distortion and the generation of unwanted harmonics. This can limit their performance in high-speed and high-power applications. The operation of Si modulators often involves changing the refractive index of the Si material using electrical signals. This can lead to heat generation, which can induce thermal effects and affect the modulator’s performance and stability [64]. Additionally, the modulation bandwidth of Si modulators can be limited by various factors, including the carrier lifetime and the device’s physical dimensions. This can restrict their performance in applications requiring high data rates.

**Figure 3:**
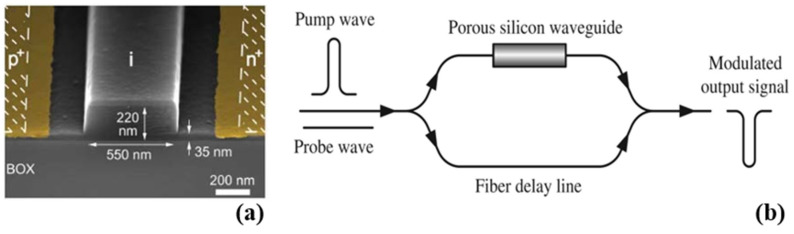
(**a**) Cross-sectional SEM image of the SOI p+-i-n+ diode nanophotonic rib WG [68]. (**b**) Schematic representation of proposed MZI device for all-optical modulation [74].

Multiple studies have documented the use of modulators based on Mach–Zehnder interferometric (MZI) designs [65,66,67,68,69,70,71,72]. A comprehensive study of an on-chip optical modulator utilizing a non-conventional Si-based platform is proposed [73]. The platform is based on the optimum design of an ultra-thin Si-on-insulator (SOI) WG, which exhibits low field confinement within the core WG and high sensitivity to the cladding index. Impressive results were obtained, with an extinction ratio (ER) exceeding 20 dB and an energy per bit of 13.21 fJ/bit for an applied voltage of 0.5 V [73]. The platform’s performance for modulation applications was found to be promising and adequate, with the added advantages of cost effectiveness and ease of fabrication. Researchers, in 2007, introduced ultra-compact Si p+-i-n+ diode Mach–Zehnder electro-optic modulators measuring 100 to 200 µm in length [68]. The active WG structure’s cross-sectional scanning electron microscope (SEM) image is presented in Figure 3a. The performance of these modulators was noteworthy, showcasing remarkable modulation efficiency. Specifically, the Vπ·L figure of merit was determined to be 0.36 V-mm. The experiment successfully demonstrated optical modulation at data rates reaching 10 Gb/s while consuming minimal RF power, with an energy consumption of merely 5 pJ/bit [68].

Another study proposed and investigated a nonlinear porous Si-based all-optical modulator using an asymmetrical MZI design [74]. The MZI comprised two couplers with an identical splitting ratio and two unbalanced arms. In this configuration, a porous Si (PS) WG is placed in only one arm of the MZI, whereas the other arm functions as a short fiber delay line, as shown in Figure 3b. The authors conducted experiments where a pulsed pump and a continuous probe wave were at the same time launched into the input port. The results indicated that the device achieved an outcome signal with approximately 14.10 dB modulation depth at the probe wavelength. The experimental conditions include an initial pulsed pump with a peak of 47.07 dB m, an extinction ratio of 10.88, and a duration of 100 ps. The continuous probe wave has a power of 0 dB m, and the PS WG is 3 mm long [74].

**Figure 4:**
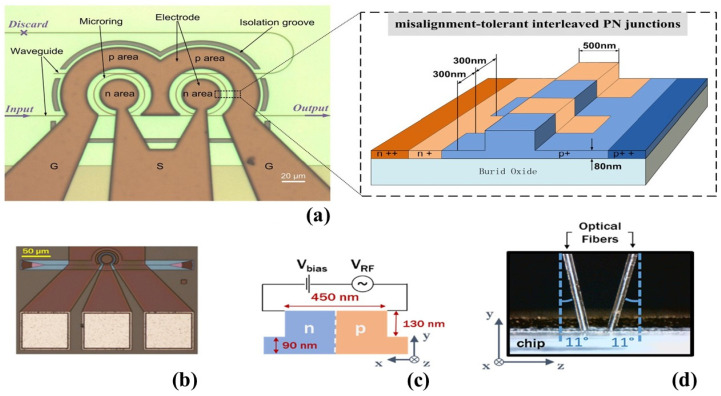
(**a**) A bird’s-eye view of the microscopic image of the manufactured cascaded micro-ring modulator and the schematic 3D view of the interleaved PN junctions [88], (**b**) RR modulator top view micrograph, (**c**) WG cross section, and (**d**) coupling scheme for the Si photonic chip [89].

Another popular type of modulator relies on ring resonators (RRs), which are actively being researched by scientists [75,76,77,78,79]. These modulators effectively manipulate light signals by leveraging the unique properties of RRs. They offer versatility and are widely utilized in photonics for various communication and scientific purposes as researchers continue to explore their potential applications [80,81,82,83,84,85,86,87]. A high-speed Si modulator based on cascaded double µ-RRs is explained in a study conducted by the authors of [88]. In Figure 4a, a top view of a microscopic picture is presented, showcasing the fabricated cascaded micro-ring modulator and a schematic 3D view of the interleaved PN junctions. The modulator achieved a modulation rate of 40 Gbit/s, with an ER of 3.9 dB [88]. To enhance the modulator’s performance, the researchers utilized a cascaded double-ring structure that enabled an ultra-high optical bandwidth of 0.41 nm, equivalent to 51 GHz [88]. In another study conducted by researchers [89], the generation of an optical comb consisting of frequency lines with extremely constant power was reported. The change in power among the frequency lines was less than 0.7 dB [89]. To achieve this, a Si RR modulator was employed. The fabrication of this structure took place on the SOI platform, utilizing processes that are compatible with CMOS technology. Figure 4b presents a top-down view micrograph of the ring, demonstrating its structure. Additionally, a diagram outlines the doped sections of the WG in a cross-sectional representation [89]. To establish connectivity with the device, grating couplers were employed, as illustrated in the micrograph. These couplers were intentionally designed to exclusively guide the TE mode within the C-band, where the mode’s primary axis aligns with the x-direction in Figure 4c. To facilitate the transmission and capture of light, two PM fibers were positioned at an 11° angle relative to the chip’s surface normal and precisely aligned with the grating couplers, as illustrated in Figure 4d. In this work, researchers characterized the ring’s complex transfer function and generated five frequency tones with a 10 GHz spacing using a dual-frequency electrical input at 10 and 20 GHz [89]. The optimal operation was observed at a small forward-bias voltage, as indicated by a comparison of comb shapes. Time domain measurements confirmed highly coherent comb signals, producing 20.3 ps-long pulses [89]. In Table 1, a comprehensive analysis is provided, showcasing the performance of different Si-based modulators that feature diverse structures.

### 4.2. Wavelength Division Multiplexing (WDM) Systems

**Figure 5:**
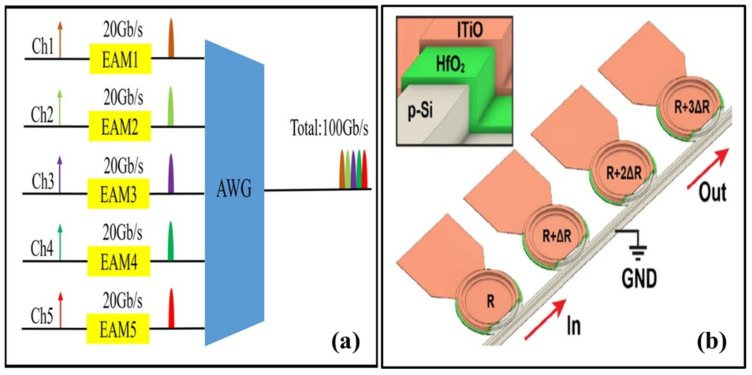
(**a**) Schematic diagram of the chip layout [108]; (**b**) 3D schematic of the on-chip WDM filters. Inset: zoom-in view of µ-RR WG with ITiO/HfO_2_/Si MOS capacitor [118].

The rapid growth of capacity demand in various interconnected systems, such as massively parallelized systems, intra-data-center networks, and wider communication networks, necessitates an urgent need to enhance the density of transceiver capacity [90]. Consequently, increasing the number of wavelength-division multiplexing (WDM) channels emerges as one of the most effective approaches to address this issue. Research and development in the field of WDM on Si photonics-integrated circuits (PICs) began nearly twenty years ago, marking the beginning of significant advancements in this area. Since then, the progress and activity in this field have remained consistently high and continue to be active even today [91,92,93,94,95,96,97,98,99,100,101,102,103,104,105,106,107]. The rise of photonic integration technology has led to a notable interest in two prominent components: arrayed WG gratings (AWGs) and micro-ring-based filters. The footprint of a Si AWG is significantly reduced thanks to the substantial index contrast between its Si core and oxide cladding. This contrast allows for efficient light confinement and manipulation within the device. Moreover, the fabrication process of Si AWGs is compatible with well-established CMOS technology, which not only facilitates the integration of AWGs with other functional devices but also leads to cost reductions. This compatibility enables large-scale production of integrated systems, providing a cost-effective solution for various applications. In a study conducted by researchers in [108], a module comprising a modulator utilizing five channels for wavelength-division multiplexing was introduced. The design of this module can be observed in Figure 5a. This module featured the integration of a Si AWG multiplexer with a channel spacing of 200 GHz and an array of electro-absorption modulators capable of achieving a data rate of 20 Gbps [108]. The results demonstrated the module’s promising capability to support transmission capacities of up to 100 Gbps, all within a compact footprint measuring 1.5 × 0.5 mm^2^ [108].

The SOI platform emerged as a highly promising avenue for realizing optical transceivers. Nevertheless, a notable drawback of the SOI platform pertained to the relatively elevated thermo-optic (TO) coefficient of Si, leading to a significant spectral response drift of approximately 90 (pm/°K) within the C-band range. This level of drift posed a considerable challenge for various applications, prompting the exploration of numerous strategies to mitigate this issue.

In their groundbreaking study, researchers introduced an innovative advancement in wavelength filters that exhibited significantly reduced sensitivity to thermal fluctuations [109]. This achievement marked the first instance of such wavelength filters, which were engineered by combining crystalline Si and hydrogenated amorphous Si (a-Si:H) WGs. The integration of these WGs took place on a common SOI substrate, facilitated by a process flow compatible with CMOS technology. To validate the efficacy of their novel concept, the team meticulously designed and manufactured MZIs and AWGs utilizing this pioneering approach. Subsequently, precise measurements were conducted to evaluate the impact of temperature variations. The results demonstrated an impressively minimal thermal drift, specifically measuring less than 1 (pm/°K) for MZIs and under 10 (pm/°K) for AWGs within the C band range [109]. This achievement not only showcases the practical viability of the proposed wavelength filters but also underscores the potential for enhanced stability in photonic applications.

Researchers have successfully demonstrated a thermally tunable add/drop filter using a long-period grating configuration that combines Si and TiO_2_ WGs [110]. The implementation of an apodized grating between the WGs achieved a high SLSR of 25.7 dB. Despite a short LPWG length of 800 µm, a narrow bandwidth of 1.4 nm was achieved due to significant group index differences between the silicon and TiO_2_ WGs. The distinct thermal coefficients of Si and TiO_2_ contributed to a 25-fold increase in the thermal dependence of the modes. A thermal tuning efficiency of 0.07 nm/mW was achieved using a thin TiN metallic heater. Additionally, cascading two LPWGs demonstrated a channel spacing of 185 GHz with low power consumption [110]. This development holds promise for DWDM applications.

The demand for optical cross-connects and reconfigurable optical add-drop multiplexers requires switches characterized by attributes such as affordability, energy efficiency, and swift sub-millisecond switching speeds. Addressing these requirements, Si emerged as a promising technology due to its capacity to integrate optical components with electronic control circuitry, enabling the development of diverse photonic devices, including switches [111,112,113]. In their study [114], researchers presented the design of a 2 × 2 switching cell, employing a thermo-optic interferometric setup with a critical component, a sub-wavelength grating. The switching cell demonstrated notable characteristics, including an extinction ratio of approximately 13 dB, insertion loss below 2 dB, and crosstalk of 12 dB across a 150 nm bandwidth. Impressively, all of this was accomplished within a remarkably compact footprint of 240 µm × 9 µm [114]. The potential application of this design in flexible telecommunication satellite payloads was showcased by employing the switching cell as a fundamental unit in an 8 × 8 dilated Banyan matrix. This configuration demonstrated impressive attributes such as a broad bandwidth (150 nm), minimal crosstalk (−38 dB), a compact footprint of around 1620 µm × 576 µm, and a relatively low power consumption at 276 mW [114]. In other work, researchers successfully demonstrated a 4 × 4 fully non-blocking crossbar switch fabric by utilizing interferometric thermal phase shifters [115]. These phase shifters employed resistive elements placed around Si WGs to achieve controlled heating. A resulting switching time of 5 μs was recorded while demonstrating energy efficiency, with a power consumption of 41 mW per individual switching element [115].

In the realm of on-chip WDM optical interconnects, Si µ-RRs exhibit remarkable capabilities as wavelength filters, offering significant benefits in terms of their ultra-compact footprint and extraordinary energy efficiency. However, it is important to note that the resonant wavelength of Si-µ-RRs is highly sensitive to both temperature variations and variations in the fabrication process [116,117]. These factors can introduce challenges when it comes to maintaining precise control over the resonant wavelength of the Si-µ-RRs. In a notable advancement, a recent experimental study [118], demonstrated gate-tuning on-chip WDM filters for the first time. The study achieved a substantial wavelength exposure across the entire channel spacing using a Si-µ-RR array (as shown in Figure 5b) that was controlled by high-mobility titanium-doped indium oxide (ITiO) gates [118]. The integrated Si-µ-RRs exhibited an unprecedented level of wavelength tunability, reaching up to 589 pm/V or VπL of 0.050 V cm while maintaining a high-quality factor of 5200 [118].

### 4.3. Photodetectors

In the realm of optical interconnect technology, the photodetector (PD) stands as a vital component responsible for converting optical signals into electrical ones. As the demand for high-capacity optical interconnect systems continues to surge, the need for high-speed PDs becomes increasingly prominent. To meet this demand, researchers have made significant strides in developing high-performance PDs using diverse absorption materials and innovative structures on the Si photonics platform. Notable examples include germanium (Ge) PDs [119,120,121], germanium tin (GeSn) PDs [122,123,124,125], heterogeneous integrated III–V PDs [3,126,127], all-Si (Si) PDs [128,129], etc. The significance of these advancements lies in their ability to facilitate efficient and reliable communication in systems that demand large data transmission capacities. High-speed PDs play a crucial role in capturing optical signals and converting them into electrical form, enabling seamless data transfer between optical and electronic domains. As the demand for faster and more efficient communication continues to grow, the development of high-speed PDs will remain a focal point of research and innovation in the field of optical interconnect.

The fabrication process of the Ge PD involves the selective growth of a Ge film onto a Si substrate. The point of this fabrication technique is to exploit the desirable characteristics of both materials. Ge is renowned for its excellent optical properties, particularly in the infrared wavelength range, which makes it highly suitable for photodetection applications. On the other hand, Si is widely recognized as a semiconductor material with superior electronic properties. In 2007, researchers presented a Ge p-i-n PD that was seamlessly incorporated into SiON and Si_3_N_4_ WGs [130]. They ensured that all the procedures and substances employed were compatible with CMOS technology and could be readily incorporated into the existing integrated circuit process technology. In later work [120], high-speed Ge p-i-n PDs for vertical incidence were introduced, featuring remarkable responsivity, and grown using reduced pressure chemical vapor deposition (CVD) [120]. The authors of [131] presented a demonstration of 100 Gbps Si-contacted Ge WG p-i-n PDs that were successfully integrated on IMEC’s Si photonics platform. In another study [132], Ge WG PDs were created on SOI substrates using selective epitaxial growth. These PDs had a width of 4 μm and were fabricated with different lengths.

**Figure 6:**
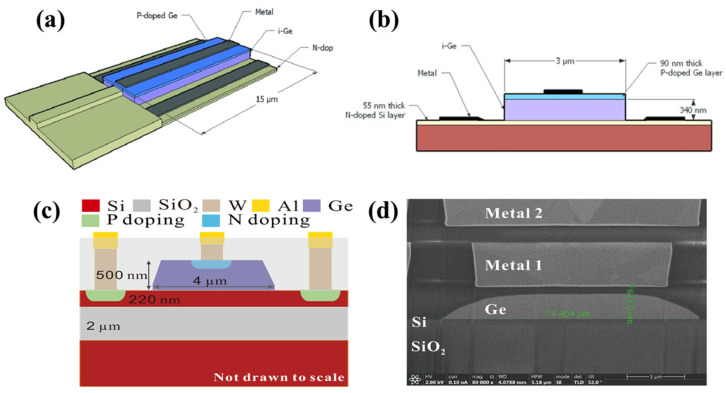
(**a**) Schematic views of pin germanium photodetector integrated into SOI WG, (**b**) cross section of the pin diode [133], (**c**) cross-sectional schematic of the fabricated vertical N(Ge)-I-P(Si) photodetector, and (**d**) SEM image of the fabricated device [134].

In a previous study [133], researchers integrated a compact pin Ge photodetector into a submicron SOI rib WG. They achieved a remarkable reduction in the detector length, which was brought down to 15 μm through a butt-coupling configuration. The schematic representations of the integration of the WG Ge PD and the cross section of the pin diode are shown in Figure 6a,b, respectively. This size reduction allowed the detector to efficiently absorb light at the specific wavelength of 1.55 μm [133]. When subjected to a 4 V reverse bias, the photodetector exhibited a −3 dB bandwidth of 42 GHz. Moreover, it demonstrated an impressive responsivity of 1 A/W at a wavelength of 1.55 μm, coupled with a low dark current density of 60 mA/cm^2^. Under a −0.5 V bias and at a slightly shorter wavelength of 1.52 μm, the photodetector still maintained a responsivity of 1 A/W [133]. In photonics-integrated circuits, the input of a regular Ge PD consisted of a single-mode Si WG, and the optical connection between the input WG and PD is linked using a tapered Si WG. Notably, a novel design featuring a multi-mode WG input PD was introduced [134]. This innovative approach allowed for the retention of high-order modes from the output end of the MM-WDMs, thereby ensuring the complete transmission of light to the detector. Figure 6c illustrates the cross-sectional architecture of the vertical N-I-P photodetector, whereas Figure 6d displays an SEM image of the manufactured device [134]. The fabricated devices exhibited low dark current (10 nA at −1 V) and high responsivity (>0.75 A/W from 1270 to 1350 nm). They showed a 23 GHz optoelectrical bandwidth at −3 V bias and achieved a clear 50 Gb/s eye diagram with NRZ modulation [134]. These results highlight the devices’ excellent performance for photodetection and high-speed optical communication.

Despite its recent successes, the Ge PD is not suitable for extended wavelength detection, such as the L band or >2000 nm, due to the rapid drop in Ge’s absorption curve beyond 1610 nm. To address this limitation, adding Sn to Ge to create a GeSn alloy is proposed as an alternative solution [122,123,124,125]. Increasing the Sn concentration decreases the direct bandgap of GeSn, resulting in an extended absorption cut-off wavelength. According to [135], researchers successfully showcased the effectiveness of a Si surface passivation technique that was compatible with CMOS technology. The initial experimental validation of a germanium–tin (GeSn) lateral p-i-n PD on a unique GeSn-on-insulator (GeSnOI) substrate was reported [136]. They achieved a cutoff detection beyond 2000 nm, with a responsivity of 0.016 A/W at the wavelength of 2004 nm [136].

**Figure 7:**
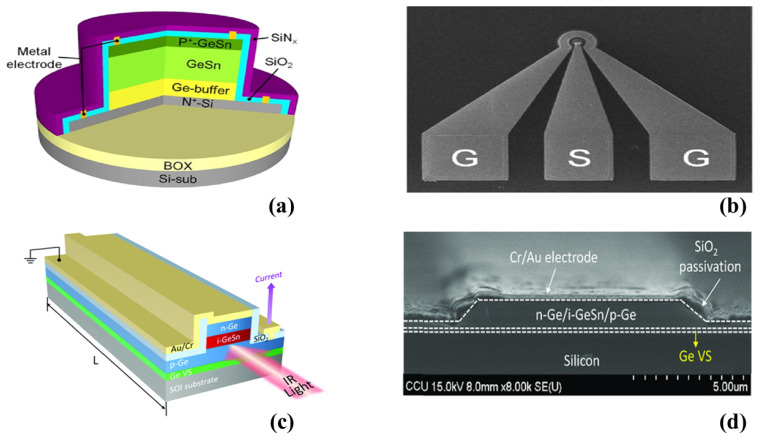
(**a**) Three-dimensional structure schematic of the photodetector, (**b**) top-view SEM image of the device [137], (**c**) schematic diagram, and (**d**) SEM image of the fabricated device [138].

In a successful demonstration [137], researchers implemented a GeSn photodetector on an SOI substrate for 2 μm wavelength applications. The 3D schematic of the normally illuminated p-i-n PD is depicted in Figure 7a, whereas Figure 7b presents the top-view SEM image of the device, featuring a mesa with a diameter of 10 μm [137]. The device exhibited a low dark current of approximately 125 mA/cm^2^ at room temperature under a reverse bias of −1 V. It also achieved an impressive optical responsivity of 14 mA/W for a 2000 nm illumination wavelength under the same bias conditions. Furthermore, the PD demonstrated a remarkable 3 dB bandwidth of around 30 GHz at −3 V [137]. A recent study demonstrated CMOS-compatible GeSn WG photodetectors (WGPDs) with a vertical p-i-n heterojunction design for operation in the 2000 nm wavelength band [138]. The schematic representation of the fabricated device is shown in Figure 7c, whereas an SEM image of the fabricated device is presented in Figure 7d. By incorporating 5.28% Sn into the GeSn active layer, the photodetection range was redshifted to 2090 nm, whereas the proposed GeSn WGPD exhibited improved light–matter interaction and optical confinement. This resulted in a responsivity of up to 0.52 A W^−1^ at room temperature within the 2000 nm wavelength band [138].

Over an extended duration, there has been extensive research on III-V PDs that exhibit exceptional performance. When compared with group IV PDs, III-V PDs demonstrate lower dark current density, allowing for improved efficiency. Furthermore, they offer wavelength tunability by employing bandgap engineering techniques, enabling the detection of a wide range of light wavelengths. Additionally, III-V PDs can achieve ultrahigh-speed operation, making them highly desirable for applications requiring rapid data processing and communication. In recent years, numerous instances have showcased the successful combination of III–V PDs with Si, employing diverse integration techniques. These methods encompass direct growth [126,139,140], wafer bonding [3,141], and transfer printing [127,142]. Each approach offers a distinct approach to the heterogenous integration of III–V PDs onto Si, resulting in significant advancements in this field. Top-illuminated PIN and modified uni-travelling carrier (MUTC) photodiodes were grown epitaxially on Si templates using InGaAs/InAlAs/InP as the material composition [143]. In this study, the achieved dark currents of the photodiodes were as low as 10 nA at 3 V, which corresponds to a low dark current density of only 0.8 mA/cm^2^. The responsivity of the photodiodes was reported to be 0.79 A/W, whereas the 3 dB bandwidth reached 9 GHz [143]. As we have already discussed, GeSn PDs extend the wavelength range of Ge PDs but face low responsivity due to lower absorption coefficients at longer wavelengths. In contrast, III–V PDs have no such limitation and easily achieve high responsivity in the short-wavelength or middle-wavelength infrared regimes. The researchers in [144], focused on the combination of InP-based type-II quantum well photodiodes on Si PICs for the range of 2 µm wavelength. In this research, the authors successfully demonstrated a responsivity of 1.2 A/W at a wavelength of 2.32 µm. The photodiodes exhibited a dark current of 12 nA at a bias voltage of −0.5 V at room temperature [144]. Comparing the transfer printed method to the direct growth method for PD production, the transfer printing approach offers the advantages of lower dark current and reduced process complexity. These benefits improve sensitivity and streamline manufacturing, making it an appealing choice for photodetector production. In a study [127], the authors proposed and demonstrated the integration of metal–semiconductor–metal (MSM) PDs based on transfer printing for use in photonic interposers. The PDs were GaAs based and operated at a wavelength of 850 nm. The larger PD exhibited a dark current of 22 nA at a bias of 2 V, whereas the smaller PD had a dark current of 7.2 nA under the same bias conditions [127]. At 2 V bias, the external responsivities for the 850 nm wavelength were measured as 0.117 A/W for the larger PD and 0.1 A/W for the smaller PD [127]. Additionally, the PDs reached a bandwidth of 20 GHz, and open-eye diagrams at a data rate of 40 Gb/s were successfully obtained [127].

All-Si PDs refer to all-Si photodetectors, which are a specific category of high-speed PDs developed within the field of Si photonics. These PDs are designed and fabricated using Si-based materials and have proven to be capable of operating at high speeds. Over time, researchers have demonstrated various types of all-Si PDs [128,129,145,146,147], showcasing the versatility and potential of this technology. The authors of study [148] reported a sub-bandgap linear-absorption-based photodetector operating in avalanche mode at a wavelength of 1550 nm. This photodetector was realized by integrating a PN diode into a Si µ-RR [148].

**Figure 8:**
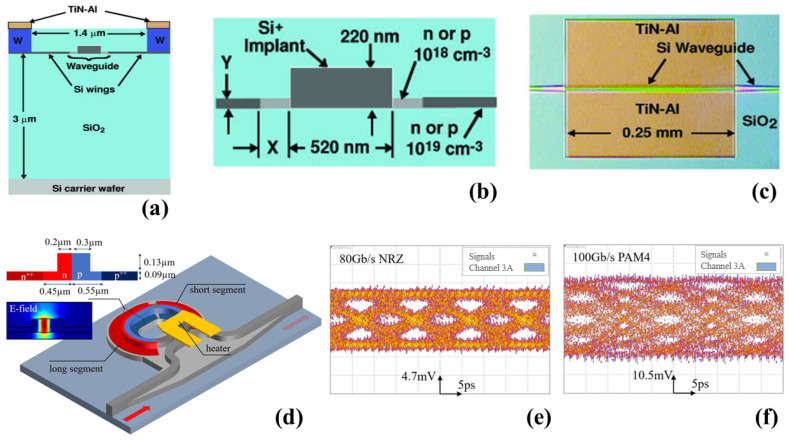
(**a**) Cross-sectional schematic of a Si WG detector, (**b**) cross-sectional schematic WG segment of the Si WG detector, (**c**) top view micrograph of Si photodetector [150], (**d**) schematic diagram of all-Si MRR APD, (**e**) eye diagrams of 80 Gb/s NRZ, and (**f**) 100 Gb/s PAM4 modulations [151].

In another study [149], the authors suggested and operated active resonance wavelength stabilization for Si µ-RRs. This was achieved by utilizing an in-resonator defect-state-absorption (DSA)-based PD specifically designed for optical interconnects [149]. The researchers conducted a demonstration of a CMOS-compatible PD based on all-Si WGs, achieving a measured speed exceeding 35 GHz [150]. The device configurations are illustrated in Figure 8a–c. To create the device, Si ions were inserted into an intrinsic Si ridge WG, which was then subjected to annealing. This PD exhibited operational capabilities within the wavelength range of 1100 to 1750 nm, with internal responsivity varying from 0.5 to 10 A/W under different bias voltages [150]. With the assistance of a cavity enhancement effect, numerous photodiodes in the past demonstrated significantly high responsivity at telecommunication wavelengths, specifically at 1310 nm. However, the underlying mechanisms that contribute to such heightened responsivity have yet to be fully understood. In a recent study [151], the researchers systematically investigated an all-Si micro-ring as a photodiode to unravel the diverse absorption processes involved [151]. The schematic representation of the all-Si MRR APD is shown in Figure 8d [151]. At a bias voltage of −6.4 V, the micro-ring exhibited a responsivity of approximately 0.53 A/W, accompanied by avalanche gain. The device further showcased a 3 dB bandwidth of around 25.5 GHz and produced open-eye diagrams with a transmission rate of up to 100 Gb/s, as shown in Figure 8e,f [151]. Table 2 provides a detailed and comprehensive analysis, offering a thorough comparison and summary of the performance of different optical photodetectors.

## 5. Si Photonics Sensors

Si photonic sensors have gained significant attention and interest in both gas sensing and biosensing applications due to their exclusive advantages, such as high sensitivity, miniaturization capabilities, and potential for integration with existing Si-based electronic systems [152]. In this section, we reviewed recent advancements in Si photonic sensors employed in biosensing and gas-sensing applications.

### 5.1. Si Photonics in Biosensing

Technological progress has brought about the convergence of various fields of science and engineering, such as electronics, medicine, and biology. This convergence has led to the development of biosensors, which are now commonly associated with point-of-care medical diagnosis and precise healthcare [153]. Biosensors have found applications in multiple areas, involving food safety, pharmaceutical research, healthcare, and cancer research [4,154]. These devices can identify and transform biological signals into electrical or optical signals [155].

Si-based materials offer significant advantages over alternative substances such as lithium and GaAs, which are commonly utilized in optical biosensors. One key advantage is their comparatively lower cost, making them more economically viable. Additionally, they can be produced on a large scale utilizing CMOS technology [156]. Moreover, Si-based materials exhibit a substantial difference in refractive index due to the distinct refractive index values of Si and Si dioxide, which are employed as the core and cladding materials, respectively. This discrepancy in the refractive index helps minimize potential errors that may arise from fluctuations in the refractive index of the sensor interface. The use of SOI wafers enables the production of both electronic and photonic components on a single chip, resulting in a compact platform consolidated on a solitary Si-based substrate [157]. In optical biosensor applications, two primary factors are consistently considered: sensitivity (*S*) and quality factor (*Q*) [158,159]. These parameters play a crucial role in distinguishing the performance of various sensors.

*S* is the measurement of the ratio between the change in sensor output and the corresponding change in the quantity being measured. It serves as an indicator of how effectively the sensor can detect and respond to variations in the measured parameter. The *S* of a biosensor is typically calculated as follows:

$$S=\frac{\Delta \lambda }{\Delta n}$$

*Q* is another essential parameter in optical biosensing. It characterizes the effectiveness and selectivity of a biosensor. A higher *Q* indicates a more precise and accurate measurement, with reduced noise and interference. The *Q* is determined by factors such as the sensor’s resonance properties, the stability of its components, and the overall system design. The *Q* is calculated by the following expression:

$$Q=\frac{\Delta \lambda \left(res.\right)}{\Delta \lambda \left(FWHM\right)}.$$

Both *S* and *Q* are crucial considerations when evaluating the performance and reliability of optical biosensors. Achieving high sensitivity and a high-quality factor is essential for accurate and precise measurements in biosensing applications.

To enhance the sensitivity of the Si-based photonic sensors, researchers have proposed highly sensitive WG structures such as suspended silicon WG [160,161,162] and subwavelength grating (SWG) WGs [163]. Suspended WGs are typically fabricated on SOI substrates, where a thin silicon layer is separated from a SiO_2_ layer by a buried oxide layer. The term “suspended” refers to the fact that the Si WG core is physically suspended above the substrate by removing the surrounding SiO_2_, thus allowing for tighter confinement of the optical mode and interaction with the surrounding environment. The suspended design allows for strong interaction between the optical mode and the analyte being sensed. This leads to increased sensitivity, making them suitable for detecting even small changes in the refractive index or other properties of the surrounding medium. Moreover, the strong confinement of light in the WG core enables compact device designs. This is particularly useful for on-chip integration with other optical components and for creating dense arrays of sensors.

SWG WGs use a periodic pattern of subwavelength-sized features to guide and manipulate light. They have gained significant attention in the field of photonics and sensing due to their unique properties and capabilities [164]. SWG WGs can be used for various applications, including sensing, due to their ability to confine and interact with light in a compact and efficient manner [165]. The periodic subwavelength structure of the WG can be tailored to achieve strong light–matter interactions with specific analytes. This enhanced interaction can enable higher sensitivities in detecting changes in refractive index, temperature, or other environmental parameters [166]. SWG WGs support the propagation of evanescent fields beyond the core region of the WG. These evanescent fields can be used to interact with substances on the WG’s surface. By monitoring changes in the evanescent field due to the presence of target molecules or changes in the surrounding medium, sensitive and label-free sensing can be achieved [167].

**Figure 9:**
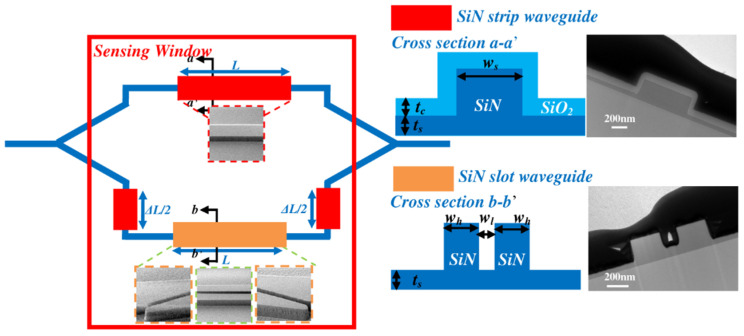
Plan-sight and cross-sectional schematic representations of the athermal MZI biosensor. Cross-sectional schematic representations and TEM figures of separate arms are also presented [177].

Extensive research has been conducted in the field of biosensors based on Si photonics, resulting in the development of several types with diverse applications [168,169,170,171,172,173,174]. These biosensors utilize the advantageous properties of Si photonics to detect and analyze biological substances effectively. The initial implementation of an interferometer WG-driven biosensing application was documented during the early 1990s [175]. This interferometer employed the Mach–Zehnder interferometer (MZI) principle, where light is divided into two separate arms and then both signals are brought together once again using a Y-shaped junction [175]. In the year 2000, the initial use of a Young interferometer (YI) as a biosensor was documented, achieving a bulk sensitivity of 9 × 10^−8^ RIU [176]. The YI employs a Y-junction to split a single optical path into two separate arms. Unlike the MZI, the interference pattern is generated outside the chip since there is no recombination of the two arms. The authors of [177] introduced a biosensor based on Si_3_N_4_ slot WGs, utilizing a Mach–Zehnder Interferometer (MZI) design, as shown in Figure 9 [177]. The biosensor was specifically engineered to exhibit minimal temperature dependency while maintaining high sensitivity and a low LoD. The measured surface sensitivity and LoD were reported to be 7.16 nm/(ngmm^−2^) and 1.30 (pgmm^−2^), respectively [177]. Additionally, the temperature dependence was found to be as low as 5.0 pm/°C. On the other hand, when water was used as the cladding material, the bulk sensitivity and LOD were reported to reach 1730(2π)/RIU and 1.29 × 10^−5^ RIU, respectively. By incorporating the Vernier effect via cascaded MZI structures, the authors achieve a sensitivity improvement factor of 8.38, resulting in a surface LoD of 0.155 (pgmm^−2^) [177].

**Figure 10:**
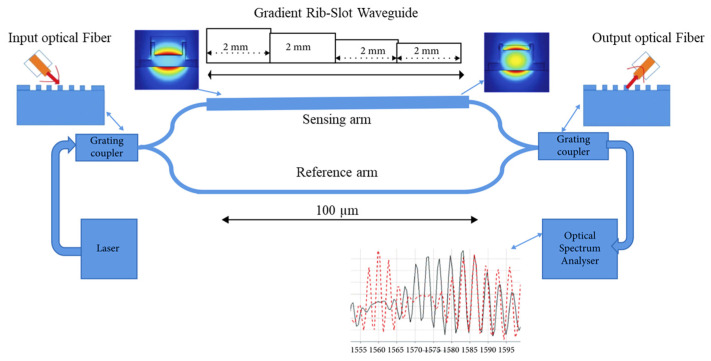
The setup of a Si_3_N_4_ biosensor with MZI and a grating coupler [179].

The efficiency of the photonic WG sensor can be validated by considering the parameter of bulk sensitivity. Current improvements in point-of-care Si photonic biosensing have led researchers to focus on identifying effective methods for improving sensitivity. One approach involves integrating a microfluidic channel made of polydimethylsiloxane (PDMS) into the sensor architecture. However, this integration may result in decreased sensitivity due to molecule leakage at the edges of the channel [178]. To address this issue, a Si_3_N_4_ MZI is utilized, which takes advantage of the refractive index of different cancer cells [179]. The authors examined the effectiveness of both gradient rib and gradient rib-slot WGs in this analysis, as depicted in Figure 10 [179]. This structure is specifically proposed to securely secure the liquid sample without the use of PDMS material. Compared with the gradient rib WG, this novel WG demonstrates significantly higher WG bulk sensitivity and device bulk sensitivity [179]. The researchers were able to achieve a WG bulk sensitivity of 2.0699 RIU/RIU and a device sensitivity of 568 nm/RIU [179].

**Figure 11:**
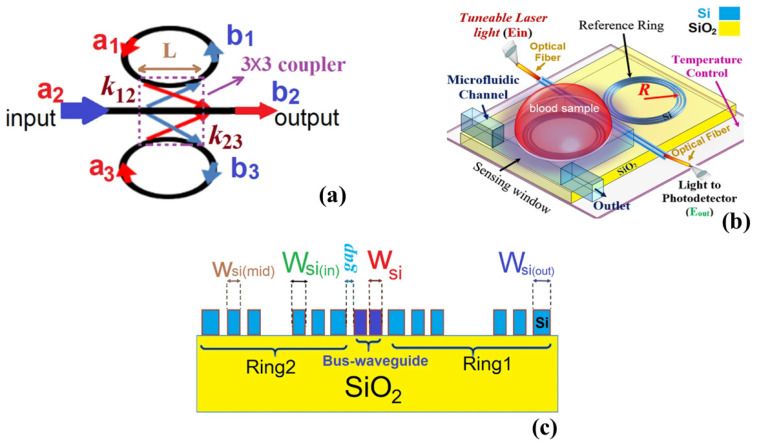
(**a**) The WG layout of an ICCRR, (**b**) 3D structure of ICCRR sensor, and (**c**) the WG cross section of the ICCRR sensor [181].

Researchers have designed a RR-based biosensor aimed at detecting cancer cells and hemoglobin concentrations while optimizing its structure to enhance *S* and *Q* [180]. The biosensor demonstrated the capability to detect a range of cancer cells and hemoglobin concentrations with a remarkable sensitivity of 200 nm/RIU and a high Q factor of approximately 2000 [180]. In a recent study conducted by researchers, an optical-based label-free biosensor was developed and optimized for sensing applications. The biosensor consisted of two indirectly coupled double-slot-WG-based µ-RRs, as shown in Figure 11 [181]. The optimized system was then employed to detect hemoglobin concentration, specifically targeting the identification of anemia disease [181]. To evaluate the biosensor’s performance, nine different concentrations of hemoglobin were introduced to the sensor for both men and women. The study aimed to determine the condition of anemia based on gender and various levels of the disease, involving normal, mild, moderate, severe, and deadly conditions [181]. Remarkably, the biosensor exhibited a high sensitivity of 1024 nm/RIU, accompanied by a minimum deflection limit of 4.88 × 10^−6^ RIU [181]. These results demonstrate the biosensor’s ability to provide precise measurements at a microscale, presenting a promising lab-on-a-chip microdevice for monitoring the health of individuals with anemia.

The application of porous Si (PSi) as an optical sensor to detect chemicals and molecular interactions has been explored. The researchers employed electrochemical etching of crystalline Si in solutions containing HF to create various PSi layers. Additionally, they employed a combination of physical, physicochemical, chemical, and electrochemical post-procedures to further enhance the properties of the PSi layers [182,183]. PhC-based biosensors have captured significant attention as a promising and groundbreaking technology. Researchers have conducted investigations into Si-based PhC devices on the SOI platform, and these devices have experienced rapid development. This technological advancement has led to the creation of various architectures, such as 1D PhC [172] and 2D PhC [184], specifically designed for the detection of biomolecules.

**Figure 12:**
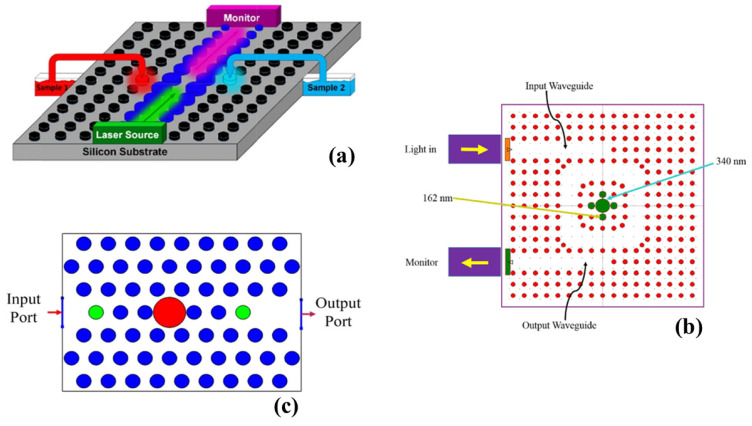
(**a**) An isometric view of the proposed structure of an optical microstructure biosensor [187]. (**b**) Schematic structure of the proposed 2D PhC biosensor [188]. (**c**) Suggested configuration for the improved design, incorporating optimized parameters [189].

In a study conducted by researchers [185], a biosensor platform was introduced that utilized microcavities coupled to an optical WG. The researchers emphasized the high sensitivity of this platform, measuring approximately 97 nm/RIU. Furthermore, the biosensor exhibited an LoD as low as 0.0055 fg [185]. In their research, the detection of the hepatitis B virus was monitored using a 1D PhC [186]. The study focused on utilizing an antibody to specifically detect the surface antigen (HBsAg-ayw). The biosensor based on the 1D-PhC design achieved an impressive LoD of 460 pg [186]. In 2021, a study was conducted to suggest a refractive index sensor capable of the detection of cancer and diabetes using PhC at the same time [187]. The fundamental structure of the proposed PhC consisted of Si rods arranged in a hexagonal lattice within an air bed. To facilitate the measurements, two tubes were utilized to accommodate the samples of cancerous or diabetic substances, as illustrated in Figure 12a [187]. The researchers recognized the importance of sensitivity and accuracy in sensor design. Consequently, they evaluated the performance of the sensor by considering the FWHM value, which was measured at 1.8 nm. Moreover, they assessed the sensitivity and FOM of the sensor following modifications made to the shape of the dielectric rods and nanocavities. The results indicated that the sensor achieved a best sensitivity value of S = 20,393 nm/RIU and an FOM of 9104.017 ± 606.93 RIU^−1^ [187]. A recent study proposed the utilization of a RR in 2D PhC biosensors [188]. The biosensor configuration, as depicted in Figure 12b, consists of an RR accompanied by two bus WGs. To form the resonator and WG, a row of rods was selectively removed from the structure. Specifically, the ring RR was composed of a large green dielectric rod encircled by four smaller rods, measuring 340 nm and 162 nm in radius, respectively [188]. The objective of this design was to enable the detection and differentiation of both normal and cancerous cells. The researchers assessed the performance of the proposed structure based on various parameters. The obtained average values for *S*, *Q*, FOM, and transmission were 308.5 (nm/RIU), 3803.55, 848.06 RIU^−1^, and 98.78%, respectively [188]. In another recent study [189], the authors introduced a biomedical sensor based on PhC, as illustrated in Figure 12c. The sensor was designed to accurately identify and differentiate between normal and abnormal brain tissues, including lesions, tumors, and cancerous tissues. Impressively, the sensor exhibited remarkable performance metrics. Specifically, it achieved a sensitivity of 1332 nm/RIU. Additionally, the sensor demonstrated an exceptionally low LoD of 9.08 × 10^−6^ and exhibited an ultra-high quality factor of 16,254 [189]. A list of some of the noteworthy proposed biosensors is presented in Table 3.

### 5.2. Si Photonics in Gas Sensing

**Figure 13:**
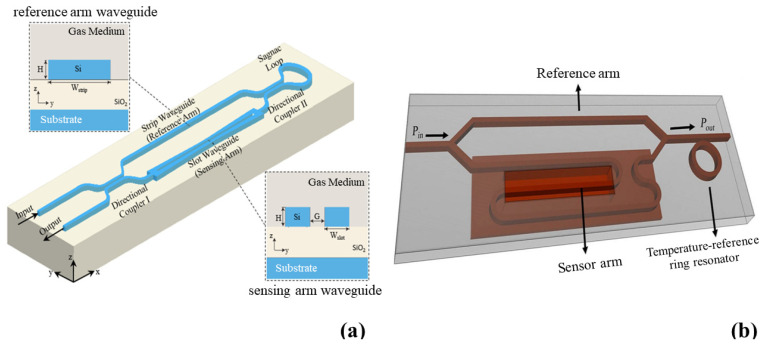
(**a**) Three-dimensional schematic representation of the LT-MZI gas sensor. Insets: cross-sectional representations of the strip (reference arm) and slot (sensing arm) WGs [203], (**b**) 3D schematic of an unbalanced MZI gas sensor supported with a temperature reference RR [204].

The significance of gas sensing has been increasingly recognized in diverse domains and for a broad spectrum of uses, including the detection of dangerous and harmful gases, inspection in industrial settings, and monitoring of the environment [190,191,192]. Numerous optical gas sensors have been created throughout time, benefiting from their remarkable attributes such as heightened sensitivity, dynamic range, stability, rapid response time, and the ability to perform multiple measurements simultaneously [193,194,195,196,197,198,199]. The ability of RI sensing to directly detect biomolecules without the need for labelling and its remarkable sensitivity to even slight variations in the surrounding medium has garnered growing attention. In 2003, an optical nanosensor utilizing Si microelectronics technology was developed; it featured an integrated MZI structure formed by total internal reflection (TIR) WGs [200]. A novel fiber MZI sensor, utilizing a single “S”-shaped fiber taper, was successfully constructed by employing nonaxial pull during the fusion splicing process [201]. The fabricated structure had a characteristic size of 660 μm in length and an axial offset of 96 μm. This particular MZI, based on the S-shaped fiber taper, demonstrated a high RI sensitivity of 1590 nm/RIU within the refractive index range of 1.409–1.425. Additionally, it exhibited a strain sensitivity of approximately −60 pm/microstrain [201]. In another study, a novel mid-infrared (MIR) gas sensor utilizing a suspended Si WG was proposed [202]. Two optimized designs for wavelength and intensity interrogation were developed, achieving high sensitivity and figure of merit [202]. In a recent study [203], researchers introduced a compact refractive index gas sensor by employing an SOI loop-terminated MZI (LT-MZI) and incorporating a slot WG in the sensing arm, as depicted in Figure 13a [203]. Despite its short sensing arm length of 150 μm, the sensor achieved a device sensitivity of 1070 nm/RIU and an impressive figure of merit (FOM) of 280.8 RIU^−1^ at a wavelength of 1.55 μm [203]. Another recent study, [204], examined the gas sensing capabilities of a compact Si photonics MZI with a coiled sensing arm, as shown in Figure 13b. The sensor, fabricated using deep UV lithography, demonstrated a sensitivity of approximately 1458 nm/RIU and an LoD of approximately 8.5 × 10^−5^ RIU when tested with helium (He) and nitrogen (N_2_) gases [204]. The sensor’s temperature sensitivity was found to be 166 pm/°C. Incorporating a cladded ring-resonator post-MZI (Figure 13b) allowed for accurate temperature compensation and resolved temperature drift due to gas flow [204].

**Figure 14:**
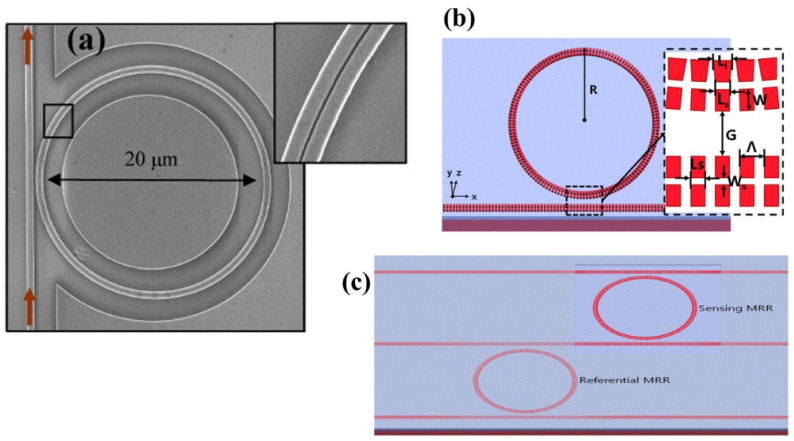
(**a**) SEM picture of a Si-slotted µ-RR. Inset shows the slot WG in the ring [209], (**b**) 3D representation of the proposed T-SWGS-µ-RR sensor, and (**c**) structural drawing of the two cascaded T-SWGS-µ-RR sensor [211].

µ-RRs in SOI structures have gained attention in sensing due to their high sensitivity to refractive index changes, wavelength multiplexing capability, and compact size [205,206,207]. µ-RRs offer precise detection of analytes, simultaneous measurement of multiple parameters, and integration into portable sensing systems, making them promising for diverse applications. In 2011, the on-chip interrogation of a gas sensor based on SOI µ-RRs was demonstrated using a compact AWG spectrometer [208]. The researchers utilized a 200 GHz SOI AWG with closely spaced output channels to analyze the response of an µ-RR sensor to ethanol vapor concentrations ranging from 100 to 1000 ppm [208]. Researchers, in [209], demonstrated a chip-scale photonic system using a slotted µ-RR cavity, as shown in Figure 14a, to perceive minute refractive index changes in acetylene gas [209]. The nanoscale slot geometry resulted in a significant interaction factor of 0.64, leading to a device sensitivity of 490 nm/RIU. By utilizing a resonator with a Q factor of 5000, they successfully detected refractive index changes at the order of 10^−4^ [209]. Recently, researchers investigated and introduced a compact grating double-slot µ-RR (GDS-µ-RR) on an SOI platform as a potential solution for achieving a refractive index sensor with a wide measurement range and high sensitivity [210]. The refractive index sensing experiments yielded a sensitivity of 433.33 nm/RIU, a Q-factor of 4325, and a DL value of 8.26 × 10^−4^ RIU [210]. The researchers concluded that the proposed compact GDS-µ-RR exhibited outstanding performance and held significant potential for various sensing applications. Another study on µ-RR, suggested a high-performance refractive index sensor utilizing a single trapezoidal subwavelength grating slot-µ-RR (T-SWGS-µ-RR)) [211]. The T-SWGS-µ-RR design shown in Figure 14b, achieved a high sensitivity of 823 nm/RIU, a large Q-factor of 2.50 × 10^4^, and a low LoD of 7.53 × 10^−5^ RIU [211]. The researchers also demonstrated an improved sensitivity of 12,151 nm/RIU and a lower LOD of 2.47 × 10^−5^ RIU by employing two cascaded T-SWGS-µ-RRs, as depicted in Figure 14c [211].

**Figure 15:**
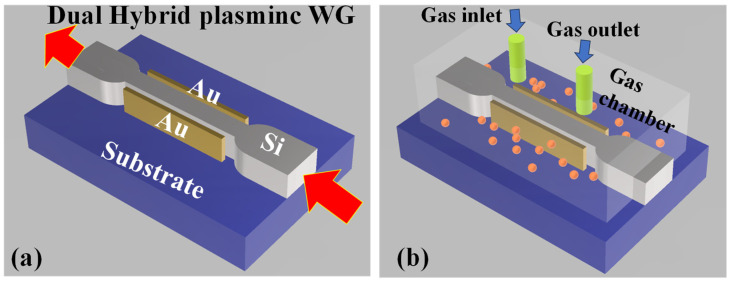
(**a**) Schematic representation of dual hybrid plasmonic WG. (**b**) Dual hybrid plasmonic WG employed for CH_4_ gas sensing. Inspired by [213].

The MIR region has become the subject of significant interest and research due to its diverse range of applications. One of the reasons the mid-IR region is particularly significant is because it holds the absorption lines of numerous important gases. Gases such as carbon dioxide (CO_2_), carbon monoxide (CO), and methane (CH_4_) [4,193,212,213] exhibit characteristic absorption patterns in the MIR range. These absorption lines correspond to specific vibrational and rotational modes of the gas molecules, allowing for their identification and quantification [214,215]. The ability to detect and measure these gases accurately is crucial for various applications, including environmental monitoring, industrial safety, atmospheric studies, and climate research. In [213], Khonina et al. achieved a substantial development in the evanescent field ratio by transforming a ridge WG into a dual hybrid plasmonic WG, as shown in Figure 15a. The WG geometry was adjusted at 3.392 μm, aligning with the absorption line of CH_4_ gas. The suggested method allowed for a larger WG cross section, facilitating flexible butt coupling of light with an augmented evanescent field. The study validated the results through finite element method (FEM) analysis and reported an elevated evanescent field ratio of 0.74 and a propagation loss of 0.7 dB/μm. A sensitivity of 0.0715 (mW/gas conc.) was also calculated by measuring the decay in transmission power due to gas absorption in the medium [213]. The schematic of the proposed gas sensing procedure is shown in Figure 15b.

**Figure 16:**
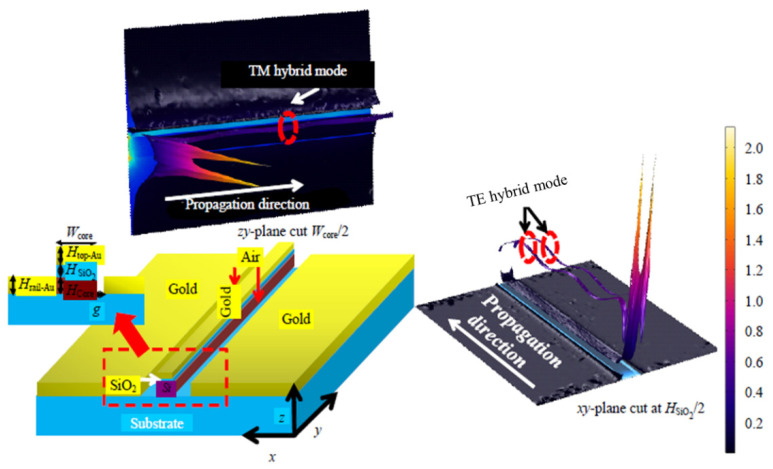
Graphical interpretation of the polarization-independent HPWG scheme. Inset exhibits the 3D E-field mapping of TE (bottom-right) and TM (top-left) polarized lights in the WG geometry [216].

In [216], Kazanskiy et al. proposed a polarization-independent strategy of a hybrid plasmonic WG (HPWG) that can be employed as an evanescent field absorption gas sensor, as illustrated in Figure 16, optimized at a wavelength of 3.392 µm for CH_4_ gas absorption [216]. The HPWG exhibited high sensitivity (S_mode_) and evanescent field ratio (EFR) for both TE and TM hybrid modes [216]. Modal analysis using the FEM confirmed the TE mode with S_mode_ = 0.94 and EFR = 0.704 and the TM mode with S_mode_ = 0.86 and EFR = 0.67. For a 20 µm-long HPWG at 60% gas concentration, both modes achieved a power dissipation of approximately 3 dB [216].

**Figure 17:**
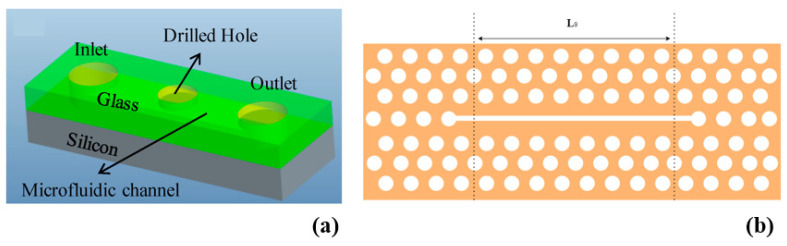
(**a**) Three-dimensional diagram of the microfluidic-based FP gas detector [220]. (**b**) Top view of L_9_ slot PhC cavity [228].

In the past, researchers developed numerous microstructured gas detectors to integrate them into micro-GC and/or microfluidic systems for rapid on-site gas detection [217,218]. Among the various detection methods, an encouraging approach relied on the utilization of the Fabry–Perot (FP) cavity. This detection scheme had gained popularity, not only in gas detection but also in other fields such as biosensing such as for temperature, strain, and humidity sensing. The FP cavity was favored due to its uncomplicated fabrication process and straightforward measurement setup. Researchers, in [219], investigated a stable optofluidic FP resonator that comprised Si cylindrical Bragg mirrors along with a central capillary tube [219]. In other work, a non-destructive and versatile microfluidic-based Fabry–Pérot (FP) gas detector (as shown in Figure 17a) was created and investigated by researchers [220]. The detector underwent testing using different analytes possessing diverse chemical and physical properties, as well as various concentrations. The experimental outcomes revealed a sensitivity of 812.5 nm/RIU and an LoD of 1.2 × 10^−6^ RIU for the detector [220].

PhC gas sensors have gained significant attention in recent years due to their high sensitivity and selectivity in detecting various gases [221,222,223,224,225,226]. These sensors are based on the principle of manipulating light propagation through a periodic arrangement of materials with different refractive indices. An investigation was conducted by researchers [227] on an air-slot photonic crystal cavity to achieve high-precision refractive index sensing. The cavity exhibited a high Q-factor of approximately 2.6 × 10^4^, and a significant overlap was observed between the resonant mode and the hollow core region. The experimental results demonstrated a sensitivity of 510 nm/RIU and an LoD below 1 × 10^−5^ RIU [227]. A series of *L_n_* slot PhC microcavities were proposed and experimentally demonstrated (in [228]) for their application as refractive index gas sensors, as shown in Figure 17b. The cavities, composed of a Si slab triangular PhC with *n* holes replaced by slots, showed exponential increases in quality factor with increasing *n*. An *L*_9_ slot PhC microcavity achieved a high-quality factor exceeding 30,000, a sensitivity of 421 nm/RIU, and an LoD below 1 × 10^−5^ RIU [228].

A comprehensive comparison and summary of the performance of various optical gas sensors can be found in Table 4. By examining the data presented in Table 4, readers can gain insights into the strengths and limitations of each sensor type, aiding in the selection and implementation of appropriate optical gas sensing technologies for specific applications.

## 6. Concluding Remarks

Si photonics is an advancing field of technology that combines optics and electronics using Si as the material platform. It focuses on the development of integrated photonic circuits that manipulate and control light signals, such as how electronic circuits manipulate electrical signals. Si photonics holds tremendous potential to revolutionize telecommunication by enabling faster, more efficient, and cost-effective data transmission and communication systems. As this technology continues to evolve, it is expected to play an increasingly crucial role in shaping the future of telecommunication networks. One of the primary applications of Si photonics in telecommunications is for high-speed, energy-efficient optical interconnects. These interconnects are used to transfer data between different components within data centers and supercomputers. Si photonics allows for the integration of high-performance optical transmitters, receivers, and WGs on a single chip, enabling faster data transfer rates and reducing power consumption compared with traditional copper-based interconnectors. Si photonics can be used to create optical amplifiers and regenerators that boost the optical signal’s strength, compensating for signal losses in long-distance optical communication. These components help maintain the quality and reach of optical signals in telecommunication networks.

Si photonics enables the development of compact and cost-effective optical transceivers used in fiberoptic communication systems. These transceivers integrate lasers, modulators, detectors, and other optical components on a Si chip. They are used for data transmission over long distances in optical networks, such as metropolitan area networks (MANs) and long-haul communications. Moreover, Si photonics provides the means to create compact and efficient wavelength division multiplexing devices, enabling higher data transmission capacity and increasing network bandwidth. Si photonic switches are also used in optical networks to route data packets efficiently. They can be reconfigured rapidly to establish various communication paths, enabling dynamic and flexible network architectures. Si photonics’ fast response times and low power consumption make it ideal for next-generation optical switches.

Additionally, Si photonic sensors leverage the unique properties of Si to interact with light and enable highly sensitive and selective detection. These sensing devices find applications in diverse fields, including environmental monitoring, healthcare, industrial process control, and more. Gas sensing devices based on the Si platform can detect and quantify the presence of specific gases in the environment. As mentioned earlier, the interaction between gas molecules and light in Si WGs can lead to changes in light properties, enabling the detection of various gases with high sensitivity. Moreover, Si photonics biosensors are designed to detect and analyze biological molecules or entities. By functionalizing the Si surface with specific biomolecule receptors (e.g., antibodies or DNA probes), these sensors can detect the binding of target biomolecules, such as proteins or DNA, leading to measurable changes in the transmitted light. Si photonics chemical sensors can also be used to detect and quantify the presence of certain chemicals or analytes. They are widely employed in environmental monitoring and industrial safety applications. The advantages of Si photonics sensors include high sensitivity, label-free detection (in the case of biosensors), compact size, and the potential for integration with existing Si-based electronic and photonic systems.

We believe that Si photonics sensors are still an active area of research and development and that ongoing advancements in materials, fabrication techniques, and device design are continuously improving their performance and expanding their range of applications. As the technology matures, Si photonics sensors are expected to play an increasingly significant role in diverse sensing applications, contributing to advancements in fields such as environmental monitoring, healthcare, and beyond.

## Figures and Tables

**Table 1:** Performance analysis of Si-based modulators employing MZI and ring structures.

Modulation Principle	Structure	E_bit_ (fJ/bit)	ER (dB)	References
Electro-refractive	MZI	13.21	20.36	[73]
Electro-optic	MZI	30.18	-	[71]
Electro-optic	MZI	-	30	[72]
Electro-optic	MZI	30	-	[70]
Carrier-depletion	Ring	-	3.9	[88]
Carrier-depletion	Ring	50	6.5	[87]
Carrier-injection	Ring	120	7	[79]
Carrier-depletion	Ring	680	8	[81]
Carrier-depletion	Ring	-	>10	[83]

**Table 2:** Performance Comparison and Summary of Different Optical Photodetectors.

Material	WL (nm)	Dark Current (nA)	Dark Current Density (mA/cm^2^)	Responsivity (A/W)	BW (GHz)	Speed (Gbps)	Refs
Ge	1550	35	-	0.81	75	64	[119]
Ge	1550	42	18.5	0.47	36	40	[120]
Ge	1550	1	5	0.82	29	50	[124]
GeSn	1887	-	73	0.017	-	-	[131]
GeSn	2000	0.0014	-	0.016	-	-	[132]
GeSn	2000	-	125	0.014	30	-	[133]
InGaAs/InAlAs/InP	1550	10	0.8	0.79	9	-	[141]
InP/InGaAs	1550	0.55	-	0.3	>40	40	[137]
All Si	1550	-	-	0.0728	~7	15	[148]
All Si	1550	-	-	0.0033	-	30	[149]
All Si	1310	-	-	0.53	25.5	100	[151]

**Table 3:** Comparative analysis of different optical biosensors.

Sensor Configuration	Sensitivity (nm/RIU)	Q-Factor	LOD	References
Si_3_N_4_-based MZI	568	-	-	[179]
Silica-based RR	200	2000	-	[180]
Si/SiO_2_ double-slot-WG, MRR	1024	-	4.88 × 10^−6^ RIU	[181]
SOI-based PhC WG, microcavity	97	-	0.0055 fg *	[185]
PhCs, nanocavities on Si	20,393	-	-	[187]
Si PhCs, RR	308.5	3803.55	-	[188]
Si-based PhCs	1332	16,254	9.08 × 10^−6^ RIU	[189]

**Table 4:** Comparative overview of cutting-edge optical sensors designed for detecting different types of gases.

Sensor	Configuration	Gas	Sensitivity (nm/RIU)	Q-Factor	LOD (RIU)	Refs.
MZI	RR-MZI	H_e_, N_2_	1458 5500 (Suspended MZI)	-	8.5 × 10^−5^	[204]
RR	RIB-slotted RR	CO_2_, CH_4_	20,600	-	3.675 × 10^−4^	[205]
RR	Slotted MRR	CH_4_, CO_2_	2308	-	-	[206]
RR	Slotted RR	CO_2_	300	-	-	[207]
RR	Slotted MRR	Acetylene	490	5000	10^−5^	[209]
SPP	PSWG	CO_2_	-	-	274.6 (Free-standing structure) 70.1 (Asymmetric structure)	[212]
PhC	Cryptophane-E-infiltrated PhC microcavity	CH_4_	363.8	12,923	-	[224]
PhC	PhC cavity	Tetrahydrofuran (THF) vapor	194	2 × 10^5^	4 × 10^−5^	[226]
PhC	PhC air-slot cavity	CO_2_, N_2_, H_e_	510	2.6 × 10^4^	1 × 10^−5^	[227]
PhC	Slot PhC microcavities	N_2_, CO_2_, H_e_	421	>3.0 × 10^4^	1 × 10^−5^	[228]

## Data Availability

Not applicable.
